# Report of Prevalent Diseases, from January 20 to March 21, 1822

**Published:** 1822-04

**Authors:** Roderick Macleod

**Affiliations:** one of the Physicians to the Westminster General Dispensary, and to the Royal Metropolitan Infirmary for Sick Children, &c. &c.


### Statistical Medicine.

### NO. I.

*Report of Prevalent Diseases, from January* 20 *to March* 21, 1822. By RODERICK MACLEOD, M.D. one of the Physicians to the Westminster General Dispensary, and to the Royal Metropolitan Infirmary for Sick Children, &c. &c.

IF we regard only the numerical lists of patients treated at public charities in different years, or different seasons of the same year, we are unavoidably led to form erroneous opinions of their comparative degrees of salubrity. The truth is, that this metropolis is so abundantly supplied with poor, many of whom always stand in need of medical assistance, and with hypochondriacs whose enjoyment is in direct proportion to the quantity of drugs they are able to procure, that vacancies no sooner occur than they are eagerly filled up; so that there are, at most public charities, always as many patients in attendance as the governors are entitled to recommend. Such, at least, has been the case at the Westminster General Dispensary, during a period of nearly five years that I have been one of the physicians to that establishment; although the seasons have differed considerably with regard to severity, and to the nature and fatality, of prevailing diseases.

This view, it is obvious, however, applies only to *general* institutions: in those set apart for specific diseases, as small-pox and fever, it does not hold good.

It is a common error, even among medical men, to regard a very mild winter as unhealthy; an opinion which receives some countenance from the circumstance that the number of admissions at public institutions, if not increased, is, at least, scarcely diminished. From the registers kept at medical institutions, we can ascertain, with accuracy, the comparative frequency of particular diseases; and, from a consideration of their nature, degree of severity, and other circumstances, independent on their number, we are led to legitimate conclusions regarding the public health. The season of 1794 was one of unusual severity; that of 1795, one of unusual mildness; and a reference to the Bills of Mortality will sufficiently show the comparative salubrity of the latter,—a circumstance particularly pointed out by Dr. HEBERDEN: and abundant evidence of a similar nature is to be found in the "Reports of the Diseases of London," published by Dr. BATEMAN. The present season has been very remarkable for its mildness, the temperature having varied but little: the greatest cold occurred on the 16th of January, when the minimum temperature was 30°, a degree of cold extremely inconsiderable, when compared with former winters. Accordingly, although we have had coughs in abundance, a considerable number of rheumatic affections, and some fevers, yet the coughs have been comparatively mild, and very seldom accompanied with inflammatory symptoms,—the rheumatism has scarcely ever assumed an acute form,—and, of the fevers, it is enough to say, no case has proved fatal. To consumptive patients, the season has been cruelly indulgent, and they have lingered through the winter with unusual obstinacy. A reference to the annexed catalogue of the diseases prevalent during the last two months, will show that the number of acute attacks has been, upon the whole, extremely small; and that no case has proved fatal which can be regarded as having derived its origin from the state of the atmosphere.

*Catalogue of Diseases, from January* 20 *to March* 21, 1822.

| | | | |
|---|---|---|---|
| Amenorrhœa | 2 | Enteritis | 1 |
| Anasarca | 1 | Enuresis | 1 |
| Apthæ | 2 | Fever, continued | 5 |
| Ascites | 3 | Do. remittent | 1 |
| Bronchitis, acute | 2 | Hepatitis, chronic | 3 |
| Do. chronic | 18 | Hæmoptysis | 2 |
| Catarrh | 8 | Hydrothorax | 3 |
| Colic Pictonum | 2 | Hydrocephalus, acutus | 3 |
| Cynanche Tonsil. | 2 | Do. chronic | 1 |
| Do. Parotidea | 2 | Hysteria | 10 |
| Diarrhœa | 3 | Icterus | 1 |
| Dropsy, general | 1 | Laryngitis, chronic | 1 |
| Dyspepsia, simple | 8 | Leucorrhœa | 2 |
| Do. with Constipation | 3 | Marasmus | 2 |
| Do. with Colic | 1 | Menorrhagia | 1 |
| Do. with Pyrosis | 1 | Nephralgia | 1 |
| Do. with Gastrodynia | 5 | Pertussis | 7 |
| Do. with Pain under short Ribs of left Side | 3 | Phthisis | 8 |
| Do. with Vomiting | 1 | Pleuritis | 1 |
| Do. with general Tremors | 1 | Pneumonia | 6 |
| Dysuria | 1 | Purpura Hæmorrhagica | 1 |
| Dyspnœa | 2 | Rhacitis | 1 |
| Determination to the Head | 9 | Rheumatism, acutus | 1 |
| Do. with Paralysis | 4 | Do. chronic | 17 |
| Do. with Incip. Cataract. | 1 | Schirrus Liver | 1 |
| Do. with Deafness | 1 | Tænia | 1 |
| Erysipelas | 1 | Varicella | 1 |
| Epilepsy | 2 | Variola | 2 |
| | | Do. *post* Vacciniam | 1 |

With regard to these cases, I have little to add to what has been already mentioned. The pneumonia was so mild as almost always to yield to the first bleeding. In the case of laryngitis, the disease has existed for four months, and is marked at present by a weak croaking voice, tenderness to pressure, and some difficulty of breathing: it appears to improve under the use of ipecacuanha and local bleeding. One of the cases of hemiplegia underwent an unexpected cure; the patient having become suddenly deranged, and affording satisfactory proof of the recovery of his strength by knocking down the person employed to take care of him. From having been obliged to drag the leg, he now walks with comparative firmness, and speaks with much more distinctness than before. Two cases of small-pox have occurred among the patients at the Westminster Dispensary; and the disease has likewise made its appearance in Mary-le-bone. The example of small-pox after vaccination was mild, and such as used to be called chicken-pox: it occurred in a child three years of age, whose younger brother was affected with the unmodified form of the disease, and had the crusts still upon him.

*Fatal Cases.*

| | | | |
|---|---|---|---|
| Hydrothorax | 1 | Marasmus | 1 |
| Phthisis | 1 | Purpura | 1 |
| Hydrocephalus acutus | 2 | Schirrus Liver | 1 |
| Convulsiones e Dentitione | 1 | | |

*Examinations after Death.**

*Hydrothorax.*—The case which, from the symptoms, was registered as hydrothorax, was not examined after death.

*Phthisis.*—The pleura pulmonalis adherent to the pleura costalis of the left side; left lung firm, tuberculated, containing vomicæ, and being throughout of a deep purple colour; right lung comparatively healthy, but containing some dark purple spots, both on the surface and in its structure.

*Hydrocephalus.*—Both these cases occurred in children between five and six years old, and run their course in from ten to twelve days. In both, the symptoms, supposed to characterize effusion, had come on some days before death; in both, similar remedies were employed. In one, the ventricles contained from two and a half to three ounces of fluid; in the other, they contained none. In both, there was appearance of increased vascularity on the surface; by which I mean, not that venous turgescence dependent, in a great measure, upon the position of the head after death, but the fluid injection of very minute vessels, such as we see in active inflammation of other serous textures. In the case where there was no water, there was a transparent gelatinous effusion, resembling very much white currant jelly, underneath the arachnoid membrane, particularly

* In conducting these, I am indebted to the assistance of the pupils at the Dispensary, and Mr. Wade, the apothecary.

perceptible in the hollows formed between the convolutions. This appearance I have frequently found both with and without effusion into the ventricles. I have no doubt that one of these cases would find a different name among those who are able to inform us, before death, of the exact seat of diseased action; but, until more definite and satisfactory diagnostics than any yet published can be found, I must content myself with calling the disease by the old name; although this is not the first example I have met with, of a disease running the exact course of water in the head, in which no water was found.

*Convulsiones e Dentitione.*—This occurred in a child about six months old: she died in a few hours, and no morbid appearances whatever could be detected.

*Marasmus.*—The subject of this case was six years of age, and had suffered for many months under the usual symptoms. During the last three or four weeks he had constant diarrhœa, and much tenderness to pressure. It was impossible to cut into the abdomen, whatever care we employed, without wounding an intestine, so completely was the whole alimentary canal glued together, and to the peritoneum lining the cavity of the abdomen.

*Purpura.*—This patient had long laboured under a cachectic state of constitution, and had large glandular swellings about the throat, with a tumor to be felt in the abdomen, for which he had taken, at different times, large quantities of mercurial medicines. About a fortnight before death, purple spots, from the size of a split pea to that of a shilling, had made their appearance on the arms, neck, and chest, accompanied with a constant oozing of blood from the gums, nostrils, and throat. On opening the abdomen, a large, flattish, smooth body was observed projecting from under the ribs on the left side, and extending to the umbilicus. Farther examination proved this to be the spleen: it had, externally, a mottled appearance, variegated with red and purple; internally, it differed little from the healthy viscus. The length was 13½ inches; the breadth, 8⅞; the weight 5 ℔. 10½ ounces. The free margin of the liver, for about two inches on the left portion of the right, and right portion of the left, lobe, was studded with little tubercular bodies, resembling very closely, in size and appearance, the venereal warts which occur on the glans penis. On the convex surface there were several purple spots, as if from echymosis; and similar appearances presented themselves in various portions of the great and small intestines: none were observed in the stomach. The mesenteric glands were much enlarged; and, on either side of the spine, communicating with the groins, was situated a chain of glandular bodies, of a deep-purple colour, which, on being cut into, exuded a viscid fluid of similar appearance. An hydatid, about the size of a hazel-nut, was imbedded in the lower part of the left kidney. The lungs were of a deep-purple colour, resembling the state of the posterior portions of these viscera when they become turgid with blood, from the position in which bodies are usually laid after death. The thyroid cartilage was nearly ossified; the internal membrane of the larynx thickened and granulated; the mucous membrane of the trachea of a deep purple, approaching to black; towards the bifurcation, the colour became less intense, and in some parts resembled the purple vesicles on the other surfaces. The right auricle and ventricle were enlarged; the whole heart very flabby, and tinged, externally, with several purple blotches: the same appearance was distinctly seen on the internal surface of the right auricle, but not in any of the other cavities. Permission was not obtained to examine the head.

*Schirrus Liver.*—This occurred, in an old woman, from the habitual use of spirituous liquors. The liver was not increased, perhaps rather diminished, in size, and in the state usually described as schirrus of that organ,—certainly very little resembling schirrus of any other. Between the integuments and the abdominal muscles were a considerable number of tumors, about the size of hazel-nuts, which, on being cut into, were found, in general, to contain curdy pus, and possessing, in other respects, the character of struma.

